# Unraveling the impact of cancer-associated fibroblasts on hypovascular pancreatic neuroendocrine tumors

**DOI:** 10.1038/s41416-023-02565-8

**Published:** 2024-02-10

**Authors:** Ting-Yu Lai, Tsai-Chen Chiang, Chih-Yuan Lee, Ting-Chun Kuo, Chien-Hui Wu, Yi-Ing Chen, Chun-Mei Hu, Manjit Maskey, Shiue-Cheng Tang, Yung-Ming Jeng, Yu-Wen Tien, Eva Y.-H. P. Lee, Wen-Hwa Lee

**Affiliations:** 1 Department of Surgery, National Taiwan University Hospital, Taipei, Taiwan; 2 Genomics Research Center, Academia Sinica, Taipei, Taiwan; 3 Department of Internal Medicine, University of Texas Medical Branch, Galveston, TX USA; 4 Department of Medical Science, National Tsing Hua University, Hsinchu, Taiwan; 5 Department of Pathology, National Taiwan University Hospital, Taipei, Taiwan; 6 Department of Biological Chemistry, University of California, Irvine, CA USA

**Keywords:** Cancer, Biomarkers

## Abstract

**Background:**

Pancreatic neuroendocrine tumors (PNETs) with low microvessel density and fibrosis often exhibit clinical aggressiveness. Given the contribution of cancer-associated fibroblasts (CAFs) to the hypovascular fibrotic stroma in pancreatic ductal adenocarcinoma, investigating whether CAFs play a similar role in PNETs becomes imperative. In this study, we investigated the involvement of CAFs in PNETs and their effects on clinical outcomes.

**Methods:**

We examined 79 clinical PNET specimens to evaluate the number and spatial distribution of α-smooth muscle actin (SMA)–positive cells, which are indicative of CAFs. Then, the findings were correlated with clinical outcomes. In vitro and in vivo experiments were conducted to assess the effects of CAFs (isolated from clinical specimens) on PNET metastasis and growth. Additionally, the role of the stromal-cell-derived factor 1 (SDF1)–AGR2 axis in mediating communication between CAFs and PNET cells was investigated.

**Results:**

αSMA-positive and platelet-derived growth factor-α–positive CAFs were detected in the hypovascular stroma of PNET specimens. A higher abundance of α-SMA-positive CAFs within the PNET stroma was significantly associated with a higher level of clinical aggressiveness. Notably, conditioned medium from PNET cells induced an inflammatory phenotype in isolated CAFs. These CAFs promoted PNET growth and metastasis. Mechanistically, PNET cells secreted interleukin-1, which induced the secretion of SDF1 from CAFs. This cascade subsequently elevated AGR2 expression in PNETs, thereby promoting tumor growth and metastasis. The downregulation of AGR2 in PNET cells effectively suppressed the CAF-mediated promotion of PNET growth and metastasis.

**Conclusion:**

CAFs drive the growth and metastasis of aggressive PNETs. The CXCR4–SDF1 axis may be a target for antistromal therapy in the treatment of PNET. This study clarifies mechanisms underlying PNET aggressiveness and may guide future therapeutic interventions targeting the tumor microenvironment.

## Background

The pancreas comprises both endocrine and exocrine cellular components. Consequently, pancreatic tumors can be of two types: pancreatic neuroendocrine tumors (PNETs), which originate from endocrine cells, and pancreatic ductal or acinar cell adenocarcinomas (PDACs), which originate from exocrine cells. PNETs primarily originate from hormone-producing cells. A substantial proportion of these tumors are nonfunctional—they do not secrete hormones. Nonfunctional PNETs typically exhibit a prolonged disease course. Patients with metastatic PNETs often have a poor prognosis [1]. PNETs account for <3% of all primary pancreatic tumors; however, an increase has been noted in the incidence of PNETs [2]. Surgery is the primary treatment modality for many PNETs [3]. However, postoperative metastasis can result in disease recurrence or progression [4]. The treatment options available for metastatic PNETs or for adjuvant care have demonstrated limited efficacy [2]. PDACs are associated with a poor prognosis: the rate of 5-year overall survival is approximately 11% [5]. Histologically, PDACs are characterized by a hypovascular and fibrotic stroma [6]. By contrast, PNETs are associated with better prognosis: the rate of 5-year overall survival is 63% [7]. Despite their classification as “indolent,” some PNETs exhibit rapid progression and resistance to aggressive treatments, resulting in survival rates as low as that noted in patients with PDAC. Histopathological criteria that are commonly used to predict clinical aggressiveness in PNETs include angioinvasion, perineural invasion, mitosis number, and proliferation (Ki67) index. However, data from the Surveillance, Epidemiology, and End Results program indicate that only lymph node and distant metastases have been established as unequivocal markers of clinical aggressiveness [8, 9]. For PNETs, studies have emphasized the importance of low microvessel density and the presence of fibrosis as valuable indicators of aggressiveness [10–12]. However, none of these studies revealed the underlying mechanisms.

Cancer-associated fibroblasts (CAFs) are activated during the progression of pancreatic cancer. These fibroblasts play crucial roles in shaping the tumor microenvironment (TME). CAFs facilitate the production of extracellular matrix proteins and ligands that promote tumor growth, metastasis, therapeutic resistance, and immune evasion and are thus promising therapeutic targets [13, 14]. Although previous attempts to deplete CAFs failed to benefit patients with PDAC [15], the discovery of heterogeneity among PDAC CAFs potentially explains the limitations of previous strategies [13, 16]. Thus, to establish a robust foundation for using CAFs as therapeutic targets, an increasing number of studies have been exploring the heterogeneity of PDAC CAFs and the mechanisms underlying crosstalk within the TME. As mentioned, the hypovascular fibrotic stroma is a key characteristic histological feature of PDACs and is responsible for the clinical aggressiveness of these tumors. These findings suggest that PNETs with a PDAC-like (hypovascular fibrotic) stroma are likely to exhibit clinical aggressiveness. Because CAFs are responsible for the development of PDACs’ hypovascular fibrotic stroma and associated clinical aggressiveness, they may play the same role in PNETs as well.

We hypothesized that, similar to their involvement in PDACs, CAFs would be involved in regulating the fibrotic hypovascular stroma and clinical aggressiveness of PNETs. To test this hypothesis, we initially conducted α-smooth muscle actin (SMA) staining in the stroma of hypovascular PNETs, which revealed the presence of α-SMA-positive cells, which are associated with clinical aggressiveness. Subsequently, CAFs were isolated from two clinical PNET specimens; these CAFs promoted the growth and metastasis of PNET cells. Mechanistically, PNET cells secrete interleukin (IL)-1, which induces an inflammatory gene expression profile in CAFs. This inflammatory profile includes the upregulation of stromal cell-derived factor 1 (SDF1), a chemokine that interacts with the C-X-C motif chemokine receptor 4 (CXCR4) receptor on PNET cells. Consequently, the expression of anterior gradient 2 (AGR2), a protein associated with tumor growth and metastasis, is upregulated, further enhancing cancer aggressiveness. Notably, the reduction of SDF1 level in CAFs through siSDF1 or the inhibition of CXCR4 through siCXCR4 or antibodies leads to a downregulation of AGR2 expression, which suppresses the growth and metastatic potential of PNET cells. Our study findings underscore the potential of antistroma therapy for hypovascular PNETs characterized by abundant α-SMA-positive cells and clinical aggressiveness.

## Methods

### Cell lines

Human PDAC CAFs (#3830) were purchased from ScienCell Research Laboratories (Carlsbad, CA, USA), and QGP-1 cells were purchased from the Health Science Research Resources Bank (Osaka, Japan). Human pancreatic endocrine tumor cells (BON-1) were obtained from University of Texas Medical Branch (Galveston, TX, USA). HS68 and WI-38 cells were purchased from the American Type Culture Collection. All cell lines were tested for mycoplasma contamination through polymerase chain reaction (PCR) and were subsequently confirmed to be free of any mycoplasma contamination.

### CAF isolation from a PNET specimen

Human CAFs were isolated from a clinical PNET specimen (Grade II; Ki67 index, 12.7%), which exhibited multiple synchronous liver metastases. First, fresh tumor tissue was harvested, sliced, and immediately digested with 1 mg/mL collagenase, 0.1 mg/mL hyaluronidase, and 20 mg/mL DNase (Sigma‒Aldrich GmbH, Hamburg, Germany) at room temperature for 60 min. Then, the digested cell suspension was strained through a 100-µm cell strainer by using the plunger of a plastic syringe. After a 3-min spinning at 300 × *g*, red blood cells were lysed for 60 s with RBC lysis buffer containing 0.15 M ammonium chloride and 10 mM sodium ethylenediaminetetraacetate in ddH_2_O. A positive selection–based magnetic-activated cell sorting technology was used (MiltenyiBiotec, Bergisch Gladbach, Germany), following the manufacturer’s instructions. The suspended cells were incubated with anti-fibroblast magnetic beads (MiltenyiBiotec) for magnetic labeling and then resuspended in separation buffer. These cells were tested for mycoplasma contamination through PCR, the results of which were negative.

### Study group

The study included patients with PNET who had undergone pancreatectomy at National Taiwan University Hospital between 2001 and 2013. Primary pancreatic tumor specimens were embedded in paraffin blocks. For inclusion in the study, the availability of complete clinical information was essential. The study protocol was approved by the Research Ethics Committee of National Taiwan University Hospital (201703131RIND).

### Quantitative PCR

Total RNA was extracted from cells or tissues by using a NucleoSpin RNA/protein isolation kit (Macherey–Nagel, Dueren, Germany). Subsequently, reverse transcription was performed using poly dT primers and a HighScriber kit (HighQu, Kraichtal, Germany) following the manufacturer’s instructions. Gene expression levels were quantified through quantitative PCR (qPCR) by using qPCRBIO SyGreen Mix (PCR Biosystems, London, UK) and a QuantStudio Real-Time qPCR instrument (Thermo Fischer Scientific, Waltham, MA, USA). All primer sequences were designed using the Primer 5.0 software and synthesized by Integrated DNA Technologies (Coralville, IA, USA).

The primer sequences used in this study are listed in Supplementary Table S6. The gene expression levels were calculated on the basis of cycle threshold (Ct) values. The results were calculated using the 2^−ΔΔCt^ method, which involved comparing the Ct values of the target genes with the those of a reference gene (*CYCLOPHILIN*).

### Immunohistochemistry and visual scoring by pathologists

For immunohistochemical (IHC) analysis, 4-µm-thick tissue sections were prepared from formalin-fixed, paraffin-embedded primary tumors. The following antibodies against human proteins were used: anti-α-SMA (clone 1A4; 1:50; DAKO, Carpinteria, CA, USA), anti-CD31 (PECAM; 1:10; BioGenex, Fremont, CA, USA); anti-AGR2 (ab227584; 1:100; Abcam, Cambridge, UK), and anti-CXCR4 (ab181020; 1:100; Abcam). The tumor proliferation index was determined by counting the number of tumor cell nuclei that reacted with the MIB-1 antibody, which is specific to the Ki67 antigen. For each case, 1000 nuclei were counted. The final result is presented in terms of the percentage of labeled nuclei in the sample, representing the Ki67 index. The pathological specimens were graded as follows by two pathologists who were blinded to clinical parameters: Grade I (<2 mitoses per 10 high-power fields [HPFs]; Ki-67 index: <3%), Grade II (2–20 mitoses per 10 HPFs; Ki-67 index: 3–20%), or Grade III (>20 mitoses per 10 HPFs; Ki-67 index: >20%).

### Three-dimensional histology

For three-dimensional (3D) histological analysis, approximately 10 × 10 × 10 mm^3^ tissue blocks were fixed in 10% formaldehyde for 2 days and then washed in phosphate-buffered saline (PBS) for 4 days at 4 °C. Next, the specimens were cut into 350-μm-thick sections by using a vibratome. The primary antibodies anti-CD31 and anti-α-SMA were used to immunolabel the tissues. To detect the immunostained structures, Alexa Fluor 647-conjugated goat anti-rabbit secondary antibody was used in combination with Alexa Fluor 546-conjugated goat anti-mouse or anti-rat secondary antibody (1:200, Invitrogen, Waltham, MA). The nuclei were stained using either propidium iodide or SYTO 16 (Invitrogen) at room temperature for 1 h.

### Cell proliferation assay

Cell proliferation was assessed through the incorporation of bromodeoxyuridine (BrdU) by using the BrdU cell proliferation assay kit (catalog number: 2752; Merck Millipore, Darmstadt, Germany) according to the manufacturer’s instructions. A soft agar colony formation assay was performed. For this, 1.5 × 10^3^ cells were seeded in each well in complete medium containing 0.35% agar, which was layered on top of a medium having the same composition but containing 0.5% agar.

### Immunofluorescence

For fluorescent imaging, CAFs isolated from primary tumor specimens were seeded onto Nunc Lab Tek II chamber slides at a density of 0.5 × 10^4^ cells per chamber. Then, the slides were washed twice with PBS and then fixed and permeabilized using methanol for 6 min. After permeabilization, the cells were washed with PBS and then incubated with appropriate primary antibodies (platelet-derived growth factor-α [PDGFRα]: #323502, BioLegend, San Diego, CA, USA; α-SMA, #IR47-146, iREAL Biotechnology, Taiwan; Synaptophysin, #MA5-16402, Invitrogen) at 4 °C for 24 h. Subsequently, secondary antibodies (Jackson ImmunoResearch, West Grove, PA, USA) were added, and the samples were mounted onto slides by using ProLong Gold antifade agent with Hoechst to detect the nuclei. All other imaging steps were performed using a LEICA SP8-X microscope, followed by image deconvolution.

### Flow cytometry

For flow cytometry, cell suspensions were stained with allophycocyanin-conjugated anti-PDGFRα antibodies (#323512; BioLegend; final concentration: 1 µg/mL) for 30 min at 4 °C. Subsequently, a cell viability dye (BD Horizon) was added to eliminate dead cells. This step was performed for 20 min at 4 °C. Flow cytometry was performed using FACSLyric (BD Biosciences). After gating for viable cells, we analyzed PDGFRα expression. Data were analyzed using FlowJo (version 10.5.2; TreeStar Inc., San Carlos, CA, USA).

### Animal experiments

All animal experiments conducted in this study were approved by the Institutional Animal Care and Use Committee of National Taiwan University Hospital (protocol number: 20160396). For the subcutaneous model, QGP-1 cells (3 × 10^5^) alone or in combination with CAFs (1 × 10^6^) were subcutaneously injected into the backs of male nonobese diabetic/severe combined immunodeficiency (SCID) mice. At least five mice were tested under each experimental condition. For the orthotopic model, 2 × 10^5^ QGP-1.Luc cells and 2 × 10^5^ BON-1 cells were orthotopically inoculated into the mice for tumor formation. For *AGR2* knockdown, luciferase-labeled *shLacZ-* or *shAGR2-2*-expressing BON-1 cells were suspended either with or without an equal number of CAFs in 30 µL of PBS and then orthotopically injected into the mice. When an animal met any of the pre-established criteria, for example, when a tumor interfered with the animal’s ability to eat, drink, or ambulate or if a 20% weight loss was observed, the experiment was concluded early for that animal. To allocate animals to the various experimental groups, an online randomization tool (available at https://www.randomizer.org/) was used. The investigators were blinded to group allocation during the experiment and outcome assessment. This blinding was maintained until the data analysis stage to minimize any potential bias in interpreting the results.

### Microarray analysis

Gene expression was analyzed using Human OneArray Plus (HOA7.1; Phalanx, Hsinchu, Taiwan). Differentially expressed genes between QGP-1 and QGP-1 incubated with CAF conditioned medium (CM) were identified on the basis of the following criteria: log2∣fold change∣ ≥1 and *P* < 0.05, log2 ratios = “NA,” and between-sample difference in intensity ≥1000.

### RNA interference

The *AGR2*-targeting shRNA-containing lentivirus was purchased from the National RNAi Core Facility, Academia Sinica (Taipei, Taiwan). The target sequences for human *AGR2* were shAGR2-1, 5′-CTCAAGTTGCTGAAGACTGAA-3′, and shAGR2-2, 5′-CCTTGAGACTTGAAACCAGAA-3′. The negative controls were shRNAs against luciferase (shLuc976) and β-galactosidase (shLacZ1339).

### SDF1 enzyme-linked immunosorbent assay

To determine the level of SDF1 in CAF supernatant, the cell supernatant was harvested and used for human CXCL12/SDF1 DuoSet enzyme-linked immunosorbent assay according to the manufacturers’ instructions (#DY350-05; R&D Systems).

### Statistical analyses

Clinical data were assessed using the chi-square test. A two-tailed Student’s *t* test was to analyze IHC data, cell proliferation, cell migration, and gene expression. Statistical analyses were performed using STATA 14 for Windows (StataCorp, College Station, TX, USA). A *P* value of <0.05 was considered to be statistically significant.

## Results

### Hypovascular PNETs with heterogeneous or no enhancement on arterial phase computed tomography or magnetic resonance imaging exhibit clinical aggressiveness

This study included 79 patients with PNET. Preoperative contrast computed tomography (CT) or magnetic resonance imaging (MRI) data as well as paraffinized tumor specimens were available for these patients. The patients’ clinicopathological characteristics are listed in Supplementary Table S1. Within our cohort, 34 tumors (43%) exhibited clinical aggressiveness, primarily because of liver metastasis (*n* = 14), lymph node metastasis (*n* = 4), or both (*n* = 16).

**Fig. 1:**
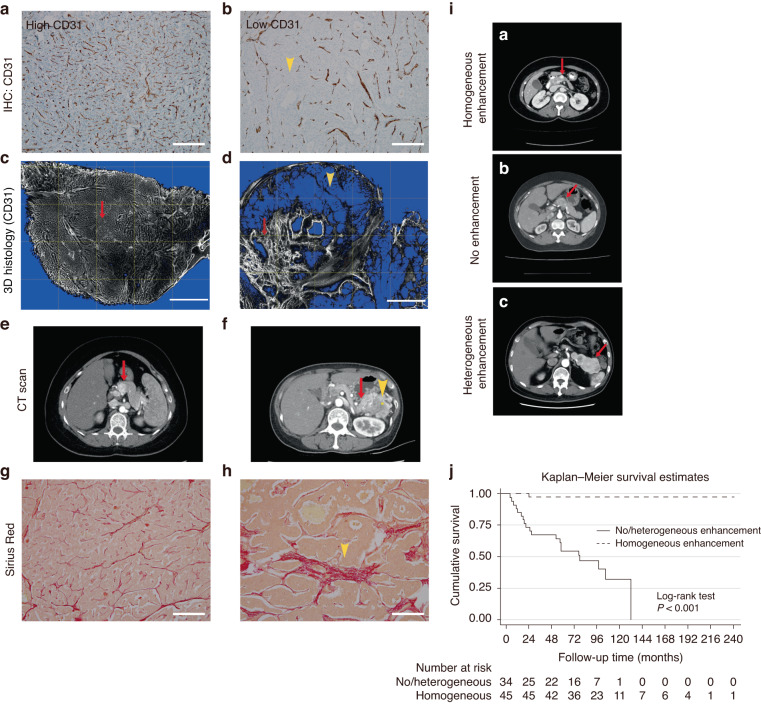
Vascularity and fibrosis of a benign PNET and an aggressive PNET. **a**, **b** Two-dimensional histology of a PNET tissue stained brown with anti-CD31 antibodies. Magnification: 100×. **c**, **d** Three-dimensional histology of a PNET tissue stained black with anti-CD31 antibodies. Magnification: 100×. **e**, **f** Images of the arterial phase contrast-enhanced abdominal CT. **g**, **h** Sirius Red staining. Magnification: 100x. The benign PNET exhibits numerous evenly distributed small-caliber vessels. **a**, **c** Contrast CT demonstrates homogeneous enhancement (red arrow in **e**), with minimal fibrous tissue (**g**). By contrast, the aggressive PNET displays heterogeneously distributed vessels. **b**, **d** Some areas exhibit only a few small-caliber vessels (yellow arrowhead), whereas other areas exhibit numerous large-caliber vessels (red arrow), in addition to heterogeneous enhancement on contrast CT (red arrow in **f**) and increased fibrous tissue (**h**). **i** Three enhancement patterns of PNETs on arterial phase CT. The red arrows indicate (**ia**) homogeneous enhancement, (**ib**) no enhancement, or (**ic**) heterogeneous enhancement. **j** Kaplan–Meier survival curves showing that patients with no or heterogeneous (solid line) enhancement on arterial phase CT or magnetic resonance imaging had significantly poorer survival than did those with homogeneous (dotted line) enhancement. CT computed tomography, PNET pancreatic neuroendocrine tumor.

To validate the hypothesis that PNETs with a stroma resembling that of PDAC would exhibit clinical aggressiveness, we performed IHC staining with anti-CD31 antibodies. Consistent with findings of Marion-Audibert et al. [10], we found two distinct distribution patterns of CD31-positive cells in the PNET specimens. Some PNETs exhibited a homogeneous distribution (Fig. 1a), whereas the others exhibited a heterogeneous distribution (Fig. 1b), making it challenging to evaluate overall vasculature by counting CD31-positive cells alone. To gain a macroscopic perspective of the tumor vasculature, we performed IHC staining for CD31 on serial sections of PNETs, reconstructing the 3D vasculature (Fig. 1c, d). The results revealed a strong correlation between tumor blood flow, evaluated through the arterial phase CT or MRI, and the intratumoral microvessel density, evaluated through 3D anti-CD31 IHC staining (Fig. 1c vs. 1e; Fig. 1d vs. 1f). PNETs with a homogeneous distribution of CD31-positive cells exhibited less fibrous tissue (Fig. 1g) than did those with a heterogeneous distribution (Fig. 1h).

For the macroscopic evaluation of the tumor vasculature, two radiologists reviewed images from the arterial phase CT and MRI enhancement scans. They assessed the tumor’s attenuation in Hounsfield Units on CT scans or signal intensity on MRI scans within an oval area of interest measuring 10 mm^2^, which was placed either within the tumor or in the adjacent pancreatic parenchyma. When assessing tumor enhancement, care was taken to avoid regions with calcifications, peritumoral pancreatitis, or adjacent normal vasculature. The relative tumor enhancement ratio was calculated by dividing a tumor’s attenuation or signal intensity by that of a healthy pancreatic parenchyma, as determined from the arterial phase. The tumors were classified as nonenhanced tumors (relative enhancement ratio <1), enhanced tumors (relative enhancement ratio ≥1), or those exhibiting both patterns. Homogeneous enhancement was characterized by all tumor regions displaying an enhancement ratio of >1, whereas heterogeneous enhancement was defined as the presence of a mixture of nonenhanced and enhanced regions within the tumor. Using the classification criteria established by d’Assignies et al. [13] and Palazzo et al. [14], we classified the 79 PNET specimens into two groups: homogeneously enhanced tumors (*n* = 45; Fig. 1ia) and nonenhanced or heterogeneously enhanced tumors (*n* = 34; Fig. 1ib, ic). PNETs with no or heterogeneous enhancement tended to be larger in size (*P* = 0.0038), have a higher tumor grade (*P* = 0.005), exhibit clinical aggressiveness (*P* < 0.0001; Supplementary Table S2), and lead to poorer overall patient survival (*P* < 0.001; Fig. 1j) than did tumors with homogeneous enhancement.

### Abundance of α-SMA-positive CAFs in PNET stroma is a significant predictor of clinical aggressiveness

**Fig. 2:**
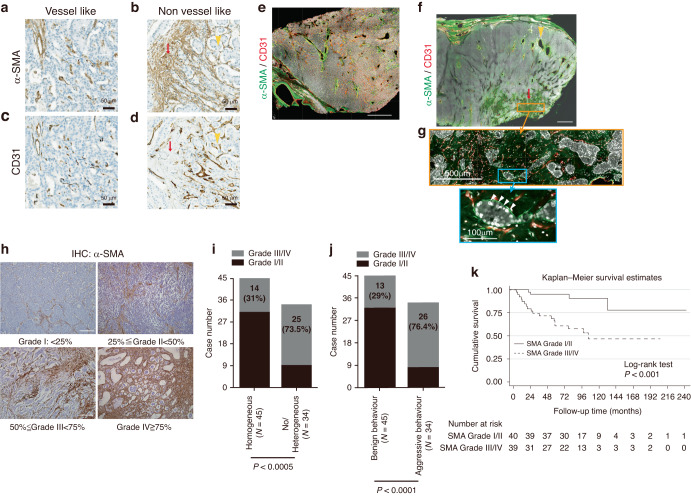
Distribution patterns of α-SMA-positive cells in a benign PNET and an aggressive PNET. **a**, **b** Two-dimensional histology of a PNET tissue stained brown with anti-α-SMA antibodies. **c**, **d** Two-dimensional histology of a PNET tissue stained brown with anti-CD31 antibodies. **e** Two-dimensional histology of serial sections of three PNETs stained with anti-α-SMA and anti-CD31 antibodies. **f**, **g** Three-dimensional histology of a PNET tissue stained with both α-SMA (green staining) and CD31 (red staining). **h** High-power field images (500x and 1000x) of the area depicted in the yellow rectangle in panel G. In the benign PNET, only a few scattered α-SMA-positive cells were observed, distributed in a vessel-like pattern (**a**, **e**). These cells colocalized with CD31-positive cells (**c**, **e**). In some areas of the aggressive PNET, scattered α-SMA-positive cells were observed in a vessel-like pattern (yellow arrowheads in **b**, **f**), which colocalized with CD31-positive cells (yellow arrowheads in **D**, **F**), whereas in other areas, numerous α-SMA-positive cells were crowded in a non-vessel-like pattern (red arrows in **b**, **f**) at the periphery of PNET cell nests, but they did not colocalize with CD31-positive cells (red arrows in **d**, **f**, **g**). On the basis of the number of α-SMA-positive cells, the 79 PNETs were classified into four grades: Grade I: <25%, Grade II: 25%–49%, Grade III: 50–74%, and Grade IV: ≥75% (**h**). PNETs with no or heterogeneous enhancement exhibited a significantly elevated number of α-SMA-positive cells (**i**). Aggressive PNETs had a significantly high number of α-SMA-positive cells (**j**). Kaplan–Meier survival curves revealed that patients with a higher proportion of α-SMA-positive cells in PNETs had significantly poorer survival (**k**). α-SMA, α-smooth actin muscle; PNET, pancreatic neuroendocrine tumor.

To investigate the potential role of CAFs in the growth and metastasis of PNETs, IHC staining for α-SMA was performed, which revealed distinct distribution patterns of α-SMA-positive cells within the TME. In some PNETs, α-SMA-positive cells were sparsely distributed in a vessel-like pattern, a pattern previously described by Marion-Audibert et al. [10] (Fig. 2a). However, in other PNETs, particularly those with no or heterogeneous contrast enhancement on cross-sectional images (CT or MRI), α-SMA-positive cells exhibited a heterogeneous distribution. This included a sparse distribution with a vessel-like pattern in certain areas (Fig. 2b, yellow arrowhead) and a dense distribution with a non-vessel-like pattern in other areas (Fig. 2b, red arrow). Through IHC staining for CD31 on serial sections of PNETs, we confirmed that in the vessel-like pattern, α-SMA-positive cells colocalized with CD31-positive endothelial cells,which indicated that these α-SMA-positive cells represent pericytes within the vessel walls (Fig. 2a, c). Conversely, in the non-vessel-like pattern, α-SMA-positive cells did not colocalize with CD31-positive endothelial cells (red arrow in Fig. 2b, d), which suggested that α-SMA-positive cells were CAFs. To obtain a macroscopic understanding of the distribution pattern of α-SMA-positive CAFs, we conducted a 3D histological analysis through the coimmunostaining of α-SMA and CD31 in two PNET samples (The tumor depicted in Fig. 2a underwent 3D histological analysis, as shown in Fig. 2e, while the tumor from Fig. 2b is presented in Fig. 2f). As shown in Fig. 2e, all α-SMA-positive cells in some PNETs were distributed in vessel-like patterns. However, as Fig. 2f, α-SMA-positive cells exhibited both vessel-like (yellow arrowhead) and non-vessel-like patterns (red arrow). In HPF views, the non-vessel-like α-SMA-positive CAFs depicted in Fig. 2f were located at the periphery of PNET cell nests (Fig. 2g).

On the basis of the positive staining rate for α-SMA, PNETs were classified into four grades (Fig. 2h): Grade I (<25%), Grade II (25%–50%), Grade III (50–75%), and Grade IV (≥75%). A higher proportion of α-SMA-positive CAFs (Grade III/IV) was frequently observed in PNETs with no or heterogeneous enhancement (25 out of 34 [73.5%]) than in those with heterogeneous enhancement (14 out of 45 [31%]; *P* < 0.0001; Fig. 2i). Furthermore, the abundance of α-SMA-positive CAFs (Grade III or IV) served as a significant predictor of clinical aggressiveness. PNETs with a high proportion of α-SMA-positive CAFs (26 out of 34 [76.4%]) were more likely to exhibit clinical aggressiveness than were those with a low proportion of α-SMA-positive CAFs (13 out of 45 [29%]; *P* < 0.0001; Fig. 2j). Moreover, patients with a higher proportion of α-SMA-positive CAFs exhibited significantly poorer overall survival (Fig. 2k). These findings indicate that mechanisms involving CAFs, independent of their association with blood vessels, contribute to the clinical aggressiveness of PNETs.

### Isolation and characterization of CAFs from a clinical specimen (Grade II PNET with synchronous liver metastases)

**Fig. 3:**
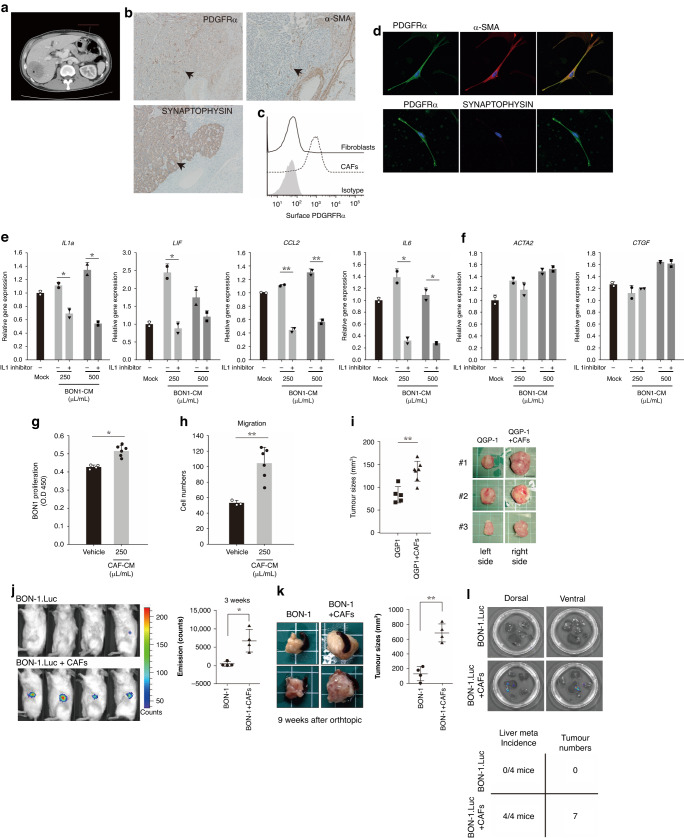
Isolation and characterization of CAFs from a clinical PNET specimen and the interaction between PNETs and fibroblasts in terms of growth and migration. **a** CT images of the PNET used for CAF isolation, displaying a hypo-enhanced tumor (arrow) in the pancreatic tail with liver metastases (*). **b** Immunohistochemical staining confirmed that the PNET was positive for synaptophysin and contained PDGFRα- and α-SMA-positive cells (arrows) (three independent experiments). **c** Representative flow cytometry histograms of surface PDGFRα-positive cells isolated from a PNET specimen (designated CAFs) and pancreatic ductal or acinar cell adenocarcinomas (designated fibroblasts) (three independent experiments). **d** Immunofluorescence images of the isolated CAFs stained for PDGFRα (green), α-SMA (red), synaptophysin (red), and DAPI (blue). Positive staining for PDGFRα and α-SMA but negative staining for synaptophysin confirmed the nature of the isolated CAFs(three independent experiments). **e** Representative bar charts displaying the quantitative polymerase chain reaction results of inflammatory CAF markers (interleukin-1a, interleukin-6, leukemia inhibitory factor, and chemokine [C-C motif] ligand 2) and (**f**) myofibroblastic CAF markers (ACTA2 and CTGF) in isolated primary CAFs treated with CM from BON-1 cells(three independent experiments). **g** A cell counting kit-8 assay for the proliferation of BON-1 cells treated with CAF CM for 48 h. **h** CAF CM promoted the migration of BON-1 cells in a Transwell assay. The results presented in (**b**–**h**) were obtained after performing at least three independent experiments(three independent experiments). **i** Tumors induced by subcutaneous inoculation of QGP-1 and CAF cells were significantly larger than those induced by QGP-1 cells alone (QGP1, *n* = 5; QGP1+CAFs, *n* = 7). **j** Three weeks after orthotopic inoculation, bioluminescence IVIS images revealed an elevated rate of tumor formation in mice that were administered both BON-1 cells and CAFs, compared to those given only BON-1 cells. (BON1, *n* = 4; BON1+CAFs, *n* = 4). **k** Nine weeks after injection, tumors induced by BON-1 cells and CAFs were significantly larger and (**l**) had more liver metastases (bioluminescence IVIS images of the mouse livers are shown as representative images) than did those induced by BON-1 cells alone (BON1, *n* = 4; BON1+CAFs, *n* = 4). Data are presented in terms of the mean + standard error of the mean values. *P < 0.05 and **P < 0.01, obtained using Student’s *t* test. CAF cancer-associated fibroblast, CM conditioned medium, PDGFRα platelet-derived growth factor-α, PNET pancreatic neuroendocrine tumor.

To investigate the characteristics of these vessel-independent α-SMA-positive CAFs, CAFs were isolated from fresh tissues obtained from two clinically aggressive PNET specimens with synchronous liver metastases and cultured (Fig. 3a). The IHC analysis confirmed the presence of synaptophysin-positive cells as well as α-SMA- and PDGFRα-positive cells in the specimens (Fig. 3b). Moreover, a comparison between the isolated CAFs and fibroblasts isolated from PDACs revealed elevated expression levels of PDGFRα in the CAFs (Fig. 3c). The purity of the isolated CAFs was further validated through immunofluorescence, and the results confirmed the expression of α-SMA and PDGFRα and the absence of synaptophysin (Fig. 3d). These findings established the purity and specific marker expression of CAFs, enabling further analysis of their functional characteristics.

### PNET cells polarize isolated CAFs toward the inflammatory phenotype through the effects of IL-1

Single-cell RNA sequencing studies have identified two primary subtypes of CAFs in the PDAC stroma: inflammatory CAFs (iCAFs) and myofibroblastic CAFs (myCAFs). Consistent with these findings, we found a considerable increase in the expression levels of iCAF markers (IL-1a, IL-6, chemokine [C-C motif] ligand 2, and leukemia inhibitory factor) but only minor changes in those of myCAF markers (ACTA2 and CTGF) when CAFs were cultured in BON-1 CM. Notably, this effect was significantly attenuated after the addition of anakinra, an inhibitor of IL-1 receptor (Fig. 3e, f). These findings strongly suggest that PNET (BON-1) cells secrete IL-1, which alters the gene expression profiles of CAFs, favoring an inflammatory phenotype.

### CAFs promote the proliferation, migration, and metastasis of PNET cells

Additional investigations were conducted to assess the effects of CAFs on the growth and metastasis of PNET cells. First, CAF CM was observed to significantly enhance the proliferation of PNET (BON-1) cells in a dose-dependent manner (Fig. 3g). Moreover, the CM promoted the migration of BON-1 cells, which indicates the involvement of CAFs in facilitating cell motility and metastatic behavior (Fig. 3h).

To further assess the role of CAFs in PNET growth, QGP-1 cells were subcutaneously injected either alone or in combination with CAFs into NOD/SCID mice. The tumors formed after the coinjection of QGP-1 cells and CAFs were significantly larger than were those formed after the injection of QGP-1 cells alone (Fig. 3i). This finding suggests that the presence of CAFs enhances the growth of PNETs in vivo. Furthermore, the role of CAFs in PNET metastasis was investigated through the orthotopical injection of BON-1 cells alone or in combination with CAFs into SCID mice. The results revealed that tumors induced by the coinjection of BON-1 cells and CAFs developed earlier (Fig. 3j), were larger in size (Fig. 3k), and exhibited higher rates of liver metastasis (Fig. 3l) than did the tumors induced by the injection of BON-1 cells alone. These results provide compelling evidence that CAFs actively promote the growth and metastatic potential of PNETs.

### Crosstalk between PNETs and CAFs

**Fig. 4:**
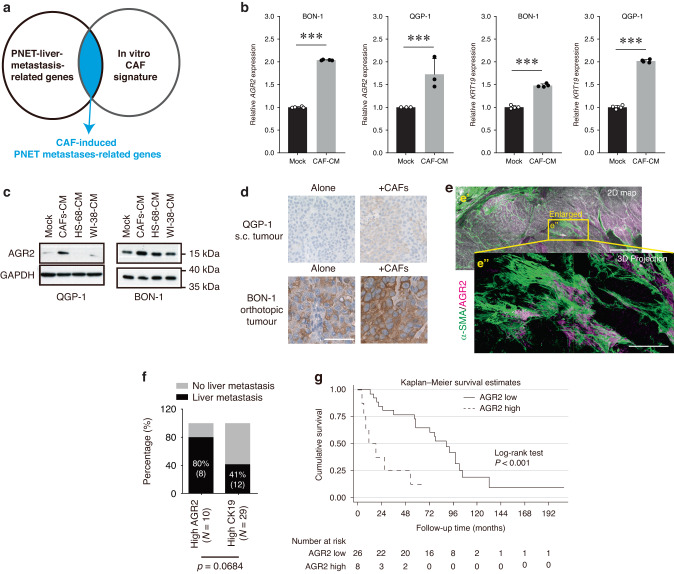
Role of AGR2 in the CAF-promotion of PNET growth and liver metastasis. **a** Analysis of the microarray data set in the Gene Expression Omnibus, which provided the gene expression data in a panel of PNETs and metastatic PNETs (refer to Supplementary Tables S2–S4). **b** CAF CM upregulated the expression of AGR2 and cytokeratin 19 mRNAs in QGP-1 and BON-1 cells (three independent experiments). Data are presented in terms of the mean + standard error of the mean values. ***P < 0.001, obtained using Student’s *t* test (**c**) CM from CAFs, but not from skin fibroblasts (HS68-CM) or lung fibroblasts (WI-38-CM), upregulated the expression of AGR2 protein in QGP-1 and BON-1 cells (three independent experiments). **d** Tumors induced by subcutaneous QGP-1 or orthotopic BON-1 cell injection plus CAF injection (Fig. 3i, k) exhibited higher expression of AGR2 (brown staining) than did those induced by QGP-1 or BON-1 cells alone. Magnification: 200x for QGP-1 cells and 400x for BON-1 cells. Scale bars: 100 and 50 µm, respectively. **e** Three-dimensional histology of a clinically aggressive PNET specimen showing the colocalization of AGR2 (opera mauve staining)- and α-SMA (green staining)-positive cells. Scale bar: 500 µm. Close association between α-SMA^+^stroma and AGR2^+^ PNET cells. *e”* is the zoomed-in view of the box in *e’*. Projection depth: 75 µm. Scale bar: 200 µm. **f** PNETs with higher expression levels of AGR2 tend to exhibit higher levels of clinical aggressiveness. A higher rate of liver metastases was observed in PNETs with a high expression level of AGR2 than in those with a high expression of cytokeratin 19. **g** Kaplan–Meier survival curves revealed poorer patient survival in individuals with aggressive PNETs exhibiting high AGR2 expression. AGER2 anterior gradient 2, CAF cancer-associated fibroblast, PNET pancreatic neuroendocrine tumor.

To identify the genes responsible for the CAF-mediated promotion of PNETs’ growth and liver metastasis, we conducted a comprehensive evaluation of the effects of CAFs on PNET cells, following a step-by-step approach. First, we assessed the effects of CAFs on PNET cells by comparing the gene expression profiles of QGP-1 cells cultured alone and those cultured in CAF CM. This analysis led to the identification the top 100 differentially expressed genes, designated as the CAF signature in QGP-1 (Supplementary Table S3). To determine the functional significance of these genes, we conducted a Gene Ontology analysis by using the DAVID Web server. This analysis revealed the involvement of the aforementioned genes in the apoptotic process and endoplasmic reticulum stress–induced unfolded protein response (Supplementary Fig. S1). Next, to identify genes specifically associated with liver metastasis in patients with PNETs, we examined the GSE73338 and GSE73339 data sets from the Gene Expression Omnibus, comparing gene expression profiles between metastatic and nonmetastatic PNETs as well as between primary and liver metastatic PNETs. By identifying common results from both data sets, we obtained a set of genes designated as PNET liver metastasis–related genes (Supplementary Table S4). Within this gene set, the genes that overlapped with the CAF signature were defined as CAF-induced PNET liver metastasis–related genes (Supplementary Table S5; Fig. 4a).

Among the CAF-induced PNET liver metastasis–related genes, AGR2 and cytokeratin 19 (CK19) were particularly analyzed because of their high fold changes (Supplementary Table S4) and potential association with tumor aggressiveness [17–20]. To validate their involvement, the effects of CAF CM on the levels AGR2 and CK19 mRNAs in QGP-1 and BON-1 cells were assessed (Fig. 4b). Additionally, the effects of CAF CM on the levels of AGR2 protein in QGP-1 and BON-1 cells were examined (Fig. 4c). Notably, only CM from CAFs, and not from skin (HS68) or lung (WI-38) fibroblasts, induced AGR2 expression in QGP-1 and BON-1 cells (Fig. 4c). To confirm these findings in vivo, IHC staining was performed for AGR2 in subcutaneous QGP-1 or orthotopic BON-1 tumors. Tumors induced by both PNET cells (QGP-1 or BON-1) and CAFs exhibited higher AGR2 expression than did those induced by PNET cells alone (Fig. 4d). A 3D histological analysis revealed the colocalization of AGR2 with α-SMA-positive cells (Fig. 4e).

To further assess the clinical relevance of AGR2 and CK19, IHC staining for AGR2 and CK19 was conducted using the 79 specimens. The extent of positive staining was graded as follows: –, <25%; +, 25–50%; ++, 50–75%; +++, >75%. PNETs with a high expression level of AGR2 (++/+++) had a higher rate of liver metastases (8 out of 10 cases) than did those with a high expression level of CK19 (12 out of 29 cases; *P* = 0.0684; Fig. 4f). A high expression level of AGR2 was found to be a significant predictor of poor overall survival in 34 patients with aggressive PNET (*P* < 0.001, Fig. 4g). Consequently, in subsequent experiments, we focused on AGR2.

### AGR2 downregulation reduces CAF-mediated promotion of PNET growth and metastasis

**Fig. 5:**
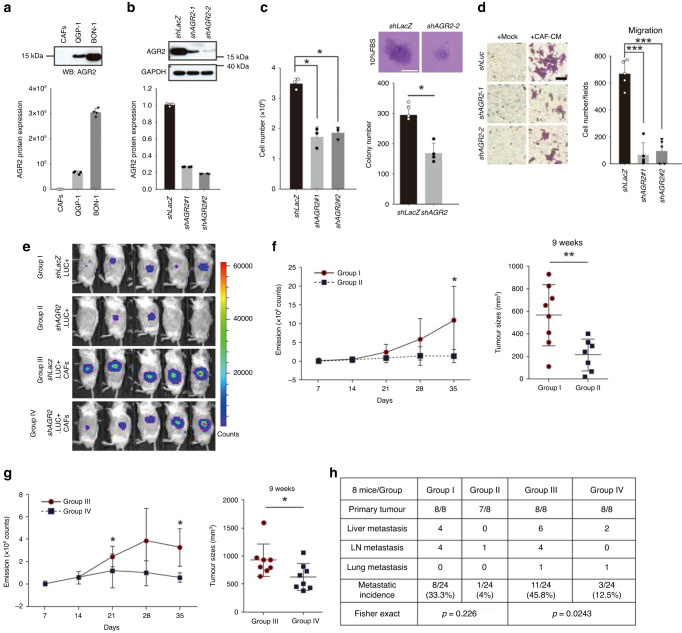
Effects of AGR2 downregulation on the growth and metastasis of PNET. **a** Immunoblotting (upper panel) and quantitative polymerase chain reaction (lower panel) revealed that PNET cells (both QGP-1 and BON-1), but not CAFs, expressed AGR2 (*n* = 4). **b** The downregulation of AGR2 protein (upper panel) and mRNA (lower panel) expression in lentiviral shRNA (*shAGR2*)–expressing BON-1 cells (*n* = 4). **c** AGR2 downregulation reduced the proliferation (left panel, *n* = 3; three independent experiments) and soft agar colony formation (right panel, *n* = 4) of BON-1 cells. **d** AGR2 downregulation also reduced the CAF-promoted migration of BON-1 cells (crystal violet staining) (*n* = 5; two independent experiments). **e** Bioluminescent IVIS images showing delayed tumor formation (after 7 days) in mice injected with *shAGR-2* BON-1 cells (Group I, *n* = 8; Group II, *n* = 8; Group III, *n* = 8; Group IV, *n* = 8). (**f** left and **g** left) Emission signal counts (tumor growth) were significantly reduced in mice injected with *shAGR2* BON-1 cells alone (**f** left panel) or with CAFs (**g** left panel). (**f** right and **g** right) Nine weeks after injection, tumor volumes were significantly lower in mice injected with AGR2-knockdown BON-1 cells alone (**f** right panel) or with CAFs (**g** right panel); bars represent the mean ± standard deviation values of tumor volumes. **h** AGR2 downregulation reduced the number of metastases in mice injected with BON-1 cells alone (*shLacZ* vs. *shAGR2*) or with CAFs (*shLacZ* + CAFs vs. *shAGR2* + CAFs). Data are presented in terms of the mean + standard error of the mean values. *P < 0.05, **P < 0.01, and ***P < 0.001, obtained using Student’s *t* test.

AGR2 mRNA and protein were expressed in both QGP-1 and BON-1 cells, but not in CAFs (Fig. 5a). To specifically downregulate AGR2 in BON-1 cells, we used a lentiviral *AGR2*shRNA, which effectively downregulated AGR2 expression (Fig. 5b). Subsequently, the functional consequences of AGR2 downregulation were investigated. The downregulation of AGR2 in BON-1 cells led to a decrease in cell proliferation (Fig. 5c) and migration (Fig. 5d). To further explore the effects of AGR2 downregulation on PNET growth and metastasis in vivo, BON-1 cells were orthotopically injected into mice. The treated mice were divided into four groups: group I, which received 2 × 10^5^ control (*shLacZ*) BON-1 cells alone; group II, which received 2 × 10^5^*shAGR2* BON-1 cells alone; group III, which received 2 × 10^5^ control (*shLacZ*) BON-1 cells in combination with 2 × 10^5^ CAFs; and group IV, which received 2 × 10^5^*shAGR2* BON-1 cells in combination with 2 × 10^5^ CAFs. Each group comprised eight mice. Tumor formation was assessed 7 days after injection by using the IVIS system. All eight mice in groups I, III, and IV exhibited detectable tumor formation, whereas only two mice in group II exhibited tumor formation (Fig. 5e). Furthermore, in postinjection weeks 2, 3, 4, and 5, the emission signal counts were significantly lower in mice injected with *AGR2*-knockdown BON-1 cells than in the control group (group II vs. group I; Fig. 5f: left panel; group IV vs. group III; Fig. 5g: left panel). After 9 weeks, the animals were sacrificed and the tumors were collected for further analysis. The primary tumor was significantly smaller (in terms of volume) in group II than in group I (*P* = 0.009; Fig. 5f: right panel) and in group IV than in group III (*P* = 0.040; Fig. 5g: right panel). Additionally, a higher number of metastases were observed in group I than in group II (*P* = 0.0226, Fig. 5h) and in group III than in group IV (*P* = 0.0243; Fig. 5h). These results indicate that the downregulation of AGR2 in BON-1 cells suppressed the growth and metastasis of tumors originating from BON-1 cells (group II vs. group I) and counteracted the positive effects of CAFs on the growth and metastasis of PNETs (group IV vs. group III).

### PNET cell–secreted IL-1 promotes the release of SDF1 from CAFs, which promote AGR2 expression in PNET cells

**Fig. 6:**
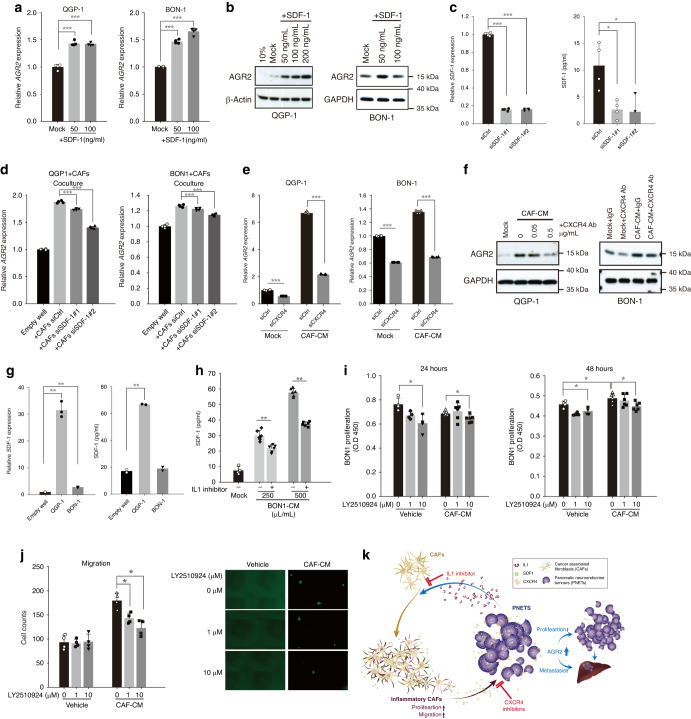
Role of CAFs in inducing AGR2 expression in PNET cells through SDF1 secretion. Quantitative polymerase chain reaction and immunoblotting revealed that SDF1 increased AGR2 mRNA (**a**) and protein (**b**) expression in QGP-1 and BON-1 cells (three independent experiments). **c** Levels of SDF1 mRNA and secreted SDF1 (ELISA) in CAFs were reduced by *SDF1*siRNAs (*siSDF1#1* and *#2*) (two independent experiments). **d** SDF1 silencing in CAFs inhibited *AGR2* expression in QGP-1 and BON-1 cells (three independent experiments). **e** Quantitative polymerase chain reaction revealed that silencing of SDF1 receptor *(CXCR4)* by siRNA (*siCXCR4*) in QGP-1 or BON-1 cells reduced the CAF-mediated expression of *AGR2* (two independent experiments). **f** Immunoblotting revealed that CXCR4 antibodies reduced the CAF-mediated expression of AGR2 in QGP-1 and BON-1 cells (three independent experiments). **g** CAFs exhibited upregulated expression of SDF1 mRNA (quantitative PCR) and protein (ELISA) when cocultured with either QGP-1 or BON-1 cells (three independent experiments). **h** Anakinra (20 μg/mL), an interleukin-1 receptor antagonist, inhibited SDF1 secretion (ELISA) from CAFs treated with BON-1 CM (three independent experiments). **i** Results of a cell counting kit-8 assay for the proliferation of BON-1 cells incubated with CAF CM treated with or without the CXCR4 antagonist LY2510924 (three independent experiments). **j** BON-1 cells cultured in CAF CM with or without CXCR4 antagonist LY2510924 were used in the Transwell assay (three independent experiments). **k** Reciprocal interactions between PNETs and CAFs promote tumor aggressiveness. PNETs secrete IL-1, which induces the expression of SDF1 in CAFs. SDF1 acts on CXCR4, thereby upregulating AGR2 expression and promoting tumor growth or metastasis. *SiSDF1*, *siCXCR4* and CXCR4 antagonist LY2510924 downregulate AGR2 expression, thereby suppressing tumor growth or metastasis. Targeting this interaction by inhibiting the CXCR4–SDF1 axis may help treat PNETs. Data are presented in terms of the mean + standard error of the mean values. *P < 0.05, **P < 0.01, and ***P < 0.001, obtained using Student’s *t* test. AGR2 anterior gradient 2, CAF cancer-associated fibroblast, CM conditioned medium, CXCR4 C-X-C motif chemokine receptor 4, ELISA enzyme-linked immunosorbent assay, SDF1 stromal-cell-derived factor 1.

To investigate the mechanisms underlying CAF-induced AGR2 expression in PNET cells, we conducted an extensive review of studies centered on soluble factors secreted by iCAFs. This investigation was conducted considering the observed shift toward inflammatory gene expression profiles in CAFs following exposure to PNET (BON-1) CM. Among the various genes affected, SDF1 (CXCL12) was demonstrated to induce AGR2 expression in glioblastoma cells [21]. Neuroendocrine tumors have been established to possess receptors for SDF1 (CXCR4) [19, 22]. Our in vitro studies further confirmed clear dose-dependent increases in the expression levels of both AGR2 mRNA (Fig. 6a) and protein (Fig. 6b) in QGP-1 and BON-1 cells when subjected to SDF1 treatment. To elucidate the specific role of SDF1, we performed knockdown experiments targeting SDF1 expression in CAFs by using SDF1-targeted siRNA (*siSDF1#1* and *siSDF1#2*; Fig. 6c). The results revealed a substantial weakening of the stimulatory effect of CAFs on AGR2 expression in both QGP-1 and BON-1 cells (Fig. 6d). Similarly, the downregulation of SDF1 receptors (CXCR4) in QGP-1 and BON-1 cells by using CXCR4-targeted siRNA (siCXCR4) led to an effective blockade of the CAF CM-induced increase in AGR2 expression (Fig. 6e). Moreover, the upregulation of AGR2 expression in QGP-1 and BON-1 cells, mediated by CAFs, was significantly attenuated when these cells were treated with an anti-CXCR4 antibody (Fig. 6f). Notably, a reciprocal interaction was observed between tumor cells and CAFs, as CM from QGP-1 or BON-1 cells stimulated the expression of SDF1 in CAFs (Fig. 6g). Furthermore, we examined the effects of IL-1 signaling inhibition on the secretion of SDF1 by CAFs. Notably, the treatment of CAFs with BON-1 CM led to 3.5- and 7-fold increases in SDF1 secretion, which were effectively countered by the use of an IL-1 receptor inhibitor (Fig. 6h).

To further investigate the effects of inhibiting the SDF1 signaling pathway by using the CXCR4 receptor on the growth and migration of PNET (BON-1) cells, we conducted additional experiments. As depicted in Fig. 6i, when BON-1 cells were incubated with CAF CM for 48 h, a noticeable increase was observed in the proliferation of BON-1 cells. However, the introduction of LY2510924, a selective CXCR4 antagonist, significantly reduced the proliferation rate of these cells (Fig. 6i). Our findings further revealed that the number of cells exhibiting invasive behavior (Transwell assay) increased by 90% when BON-1 cells were cultured in CAF CM compared with the number noted when the cells were cultured in control CM. However, this effect was dose-dependently attenuated by the LY2510924 (Fig. 6j). These findings strongly suggest that the secretion of SDF1 by CAFs stimulates the expression of AGR2 in PNET cells, consequently leading to increased proliferation and migration. Notably, this effect can be countered by using an SDF1 receptor antagonist (Fig. 6k).

## Discussion

Our study confirmed a previous finding that hypovascular PNETs with a stroma resembling PDAC exhibit clinical aggressiveness. Histologically, we observed the abundance of α-SMA-positive CAFs in the stroma of hypovascular PNETs, even in areas without visible blood vessels. The proportion of these cells was found to be a strong predictor of clinical aggressiveness and poor survival. We successfully isolated and cultured CAFs from a clinical PNET specimen. When the isolated CAFs exhibited a shift toward an inflammatory profile when they were exposed to IL-1 in the CM from PNET cells. Both in vitro and in vivo experiments demonstrated that isolated CAFs promoted the growth and metastasis of PNETs, indicating that targeting the interactions between CAFs and PNET cells can help inhibit the growth and metastasis of these tumors.

The role of CAFs in the growth and metastasis of aggressive PNETs remains largely unexplored. In a study by Marion-Audibert et al. [10] α-SMA-positive cells were exclusively observed along intratumoral capillary vessels and small arterioles within the PNET stroma. However, our findings indicated the presence of numerous vessel-independent α-SMA-positive CAFs in the hypovascular PNET stroma. Our observations align with those of Erkan et al. [23] who demonstrated a strong correlation between the abundance of α-SMA-positive CAFs in PDACs and an unfavorable prognosis of the disease. To the best of our knowledge, our study is the first to report the successful isolation and culture of CAFs from clinical PNET specimens. The isolated CAFs promoted the growth and metastasis of PNET cells, thus supporting our hypothesis that CAFs in the hypovascular and fibrotic (PDAC-like) stroma of PNETs contribute to tumor progression and clinical aggressiveness.

A recent single-cell RNA sequencing study identified three distinct CAF types in PNETs: iCAFs, myCAFs, and antigen-presenting CAFs [24]. CM from PDAC cells has been demonstrated to induce an inflammatory gene expression profile in CAFs, a phenomenon that is mitigated by anti-IL-1 and/or anti-TNF-α [13]. Similarly, we observed that CM from PNET cells induces an inflammatory gene expression profile in CAFs and that this effect can be mitigated by anti-IL-1 but not anti-TNF-α. This suggests that despite the similarities in the effects of CAFs on the growth and metastasis of PNETs and PDACs, the underlying mechanisms may vary slightly. Further research is warranted to fully understand these distinctions. Nevertheless, our study highlights the reciprocal interactions between isolated CAFs and PNET cells that closely resemble those observed between CAFs and PDAC cells, thus supporting our hypothesis that CAFs present in the hypovascular stroma of PNETs contribute to tumor progression and clinical aggressiveness, similar to their effects on PDAC.

AGR2 mediates cellular resistance to endoplasmic reticulum stress, which can lead to the upregulation of this protein [25]. Our study confirmed AGR2 as a CAF-induced PNET metastasis–related gene. Dumartin et al. also reported that AGR2 expression promotes the dissemination of PDACs [26]. Notably, we observed that the inhibition of SDF1 signaling effectively reversed the CAF-mediated promotion of PNET growth and metastasis. These results are consistent with those of Xu et al. [21], who investigated the involvement of AGR2 in SDF1-induced metastasis and its reversal following the downregulation of AGR2 expression. Our findings indicate that the SDF1 receptor on cell surfaces can serve as a therapeutic target, offering new strategies for the management of hypovascular PNETs characterized by the abundance of α-SMA-positive cells.

Numerous studies have reported that CAFs, through various mechanisms, contribute to tumor resistance to radiotherapy [27], immunosuppressive microenvironment formation [28], and resistance to chemotherapy [29]. As mentioned, the SDF1–AGR2 axis is one of the pathways associated with CAF-induced PNET liver metastasis. Additional investigations into other pathways may lead to the discovery of new targets for the early detection and therapeutic management of aggressive PNETs.

In conclusion, the present study proposes a mechanism through which CAFs contribute to the growth and metastasis of PNETs (Fig. 6k). PNET cells secrete IL-1, which induces an inflammatory gene expression profile in CAFs, characterized by the upregulation of SDF1—a chemokine that acts on the CXCR4 receptor present on PNET cells. Consequently, the expression of AGR2, a protein associated with tumor growth and metastasis, is upregulated, which further enhances cancer aggressiveness. Notably, interventions targeting the CXCR4–SDF1 axis, such as *siSDF1*, *siCXCR4*, or antibodies, can downregulate the expression of AGR2, thus suppressing the growth and metastatic potential of PNET cells. Our study underscores the potential of antistroma therapy as a treatment approach for hypovascular PNETs characterized by abundant α-SMA-positive cells and clinical aggressiveness. However, further investigations are needed to determine the effectiveness of targeting the CXCR4–SDF1 axis in antistroma therapy for aggressive PNETs.

### Supplementary information

Supplementary information

Supplementary Figure 1

## Data Availability

The data and materials that support the findings of this study are available from the corresponding author upon reasonable request.
