# Supplementary material for: Unraveling the impact of cancer-associated fibroblasts on hypovascular pancreatic neuroendocrine tumors
Source: Br J Cancer. 2024 Feb 10;130(7):1096–108. doi: 10.1038/s41416-023-02565-8 (PMC10991442; doi:10.1038/s41416-023-02565-8)
Supplement: Supplementary file 1 — Supplementary information [file 41416_2023_2565_MOESM1_ESM.docx]

**SUPPLEMENTARY FIGURE LEGEND**

**Fig. S1** GO analysis revealed that differentially expressed genes were associated with the apoptotic process and endoplasmic reticulum unfolded protein response (Supplementary Fig. S1).

**Table S1.** Demographical information for 79 studied patients**.**

|  | **All**  **N= 79** | **Aggressive behavior**  **N=34** | **Benign behavior**  **N=45** | **P-value** |
| --- | --- | --- | --- | --- |
| Age (years), mean ± SD | 53.9 ± 13.6 | 53.0 ± 12.9 | 54.5 ± 13.7 | 0.6000 |
| Sex, male/female | 38/41 | 18/16 | 20/25 | 0.4542 |
| Functional, yes/no | 5/74 | 1/33 | 4/41 | 0.2824 |
| Symptoms, yes/no | 33/46 | 22/12 | 11/34 | 0.0003 |
| Head/(neck/body/tail) | 33/46 | 17/17 | 16/29 | 0.1974 |
| Tumor size (cm), mean ± SD | 4.1 ± 3.4 | 5.8 ± 3.9 | 2.84 ± 2.4 | 0.0002 |
| LN metastases, yes/no | 20/59 | 20/14 | 0/45 | < 0.0001 |
| Grade, 1/2/3 | 48/26/5 | 11/18/5 | 37/8/0 | < 0.0001 |
| Perineural invasion, yes/no | 24/55 | 21/13 | 3/42 | < 0.0001 |
| Lymphovascular invasion, yes/no | 37/42 | 27/7 | 10/35 | < 0.0001 |
| Homogenous enhancement/No or heterogeneous enhancement on CT and/or MRI | 45/34 | 4/30 | 41/4 | < 0.0001 |
| Distribution patterns of α-SMA-positive cells, vessel-like/non-vessel-like | 37/42 | 9/25 | 28/17 | 0.002 |
| Degree of α-SMA staining, low (+, ++)/high (+++, ++++) | 40/39 | 8/26 | 32/13 | < 0.0001 |
| AGR2 expression, low (-, +)/high (++, +++) | 69/10 | 26/8 | 43/2 | 0.0115 |
| Cell membrane expression of CXCR4, yes/no | 17 /61 | 11/22 | 6/39 | 0.035 |

Functional PNETs were defined as the tumors' secretions lead to clinical symptoms such as hypoglycemia or peptic ulcer.

**Table S2 (related to Fig. 1I).** Comparison of the clinicopathological characteristics between pancreatic neuroendocrine tumors (PNETs) with homogeneous vs. no or heterogeneous enhancement.

|  | **All**  **N=79** | **Homogeneous**  **enhancement**  **N=45** | **No or heterogeneous enhancement N=34** | **P-value** |
| --- | --- | --- | --- | --- |
| Age (years), mean ± SD | 53.9 ± 13.6 | 54.1 ± 13.5 | 53.6 ± 13.8 | 0.9000 |
| Sex, male/female | 38 / 41 | 19 / 26 | 19 / 15 | 0.2289 |
| Functional, yes **/** no | 5 / 74 | 4 / 41 | 1 / 33 | 0.2824 |
| Symptoms, yes **/** no | 33 / 46 | 12 / 33 | 18 / 16 | 0.0172 |
| Location, proximal* **/** distal^#^ | 33 / 46 | 17 / 28 | 16 / 18 | 0.4076 |
| Tumor size (cm), mean ± SD | 4.1 ± 3.4 | 3.1 ± 2.9 | 5.4 ± 3.6 | 0.0038 |
| Lymph node metastases, yes **/** no | 18 / 61 | 2 / 43 | 16 / 18 | < 0.0001 |
| Grade, 1/2/3 | 48 / 26 / 5 | 35 / 10 / 0 | 13 / 16 / 5 | 0.0005 |
| Liver metastases, yes **/** no | 30 / 49 | 2 / 43 | 28 / 6 | < 0.0001 |
| Aggressive clinical behavior,  yes **/** no | 34 / 45 | 4 / 41 | 30 / 4 | < 0.0001 |
| Distribution patterns of α-SMA-positive cells, vessel-like **/** non-vessel-like | 37 / 42 | 29 / 16 | 8 / 26 | < 0.0001 |
| Amount of α-SMA-positive cells, (I, II) **/** (III, IV) | 40 / 39 | 31 / 14 | 9 / 25 | < 0.0001 |

*, head, uncinate process.

#, neck, body, tail.

**Table S3 (related to Fig. 4A).** Effects of cancer-associated fibroblasts (CAFs) isolated PNET on the gene expression of QGP-1 PNET cells obtained by comparing the gene expression profiles of QGP-1 cells cultured alone and those co-cultured with CAFs. The top 100 genes differentially expressed genes are listed below and designated as the fibroblast signature.

| **Up-regulated genes** | | | **Down-regulated genes** | | |
| --- | --- | --- | --- | --- | --- |
| **Gene** | **Ratio(log)** | **P-value** | **Gene** | **Ratio(log)** | **P-value** |
| *UBE2G1* | 4.19 | 1.75E-17 | *FST* | -2.81 | 5.71E-11 |
| *SFRP1* | 4.08 | 3.69E-04 | *CRISP2* | -2.36 | 7.83E-09 |
| *AREG* | 2.96 | 1.49E-06 | *BLM* | -2.28 | 1.50E-05 |
| *S100A2* | 2.92 | 7.91E-23 | *NKX2-2* | -1.98 | 1.21E-04 |
| *INHBA* | 2.72 | 9.24E-19 | *SLC12A5* | -1.95 | 8.40E-04 |
| *GEM* | 2.71 | 1.21E-08 | *CA8* | -1.93 | 2.02E-15 |
| *GP2* | 2.53 | 5.57E-10 | *BMPER* | -1.93 | 9.48E-05 |
| *MMP1* | 2.51 | 6.16E-08 | *PCSK2* | -1.85 | 8.09E-07 |
| *C2CD4A* | 2.48 | 1.44E-11 | *SPAG17* | -1.83 | 4.34E-12 |
| *ANXA2P1\|ANXA2\|ANXA2P3* | 2.46 | 2.20E-18 | *MAP6* | -1.78 | 5.98E-03 |
| *ID1* | 2.30 | 5.77E-12 | *ANTXR1* | -1.76 | 1.49E-09 |
| *HIST1H2BO* | 2.27 | 1.41E-10 | *HEPACAM2* | -1.68 | 6.49E-04 |
| *DCLK1* | 2.27 | 2.68E-04 | *ZNF860* | -1.66 | 2.14E-05 |
| *SQSTM1* | 2.27 | 1.23E-18 | *RC3H1* | -1.65 | 8.61E-05 |
| *FAM84A* | 2.19 | 9.50E-16 | *MAFB* | -1.63 | 2.12E-02 |
| *DUSP5* | 2.18 | 3.65E-13 | *FZD1* | -1.62 | 5.90E-16 |
| *ATF3* | 2.17 | 7.46E-11 | *APBA2* | -1.61 | 6.55E-04 |
| *LILRA2* | 2.15 | 2.39E-14 | *GUCY1A3* | -1.57 | 1.21E-06 |
| *LAMP3* | 2.15 | 9.49E-10 | *OR5AN1* | -1.57 | 3.94E-02 |
| *DDIT4* | 2.12 | 3.04E-07 | *TMEM170B* | -1.54 | 4.94E-09 |
| *ECE2* | 2.09 | 7.54E-09 | *INSR* | -1.53 | 2.95E-02 |
| *PAEP* | 2.07 | 9.09E-03 | *KCNB1* | -1.52 | 2.22E-06 |
| *TRIB3* | 2.04 | 1.67E-06 | *TNFRSF14* | -1.5 | 6.33E-03 |
| *S100A11* | 2.02 | 3.80E-10 | *NEU4* | -1.49 | 1.22E-02 |
| *OSCAR* | 2.01 | 3.57E-02 | *SUSD4* | -1.48 | 1.26E-10 |
| *ECM1* | 2.01 | 5.78E-09 | *ST3GAL6* | -1.46 | 1.23E-05 |
| *KRT6A* | 2.00 | 1.37E-06 | *LFNG* | -1.45 | 1.04E-05 |
| *HTN3* | 1.98 | 3.69E-02 | *MACROD2* | -1.44 | 8.42E-05 |
| *OR51Q1* | 1.98 | 3.59E-04 | *CA10* | -1.43 | 2.97E-03 |
| *PTHLH* | 1.98 | 1.08E-07 | *GRAMD1C* | -1.42 | 6.71E-08 |
| *INHBE* | 1.96 | 2.51E-07 | *SLC7A7* | -1.41 | 6.81E-04 |
| *S100A10* | 1.96 | 2.44E-12 | *YPEL3* | -1.4 | 1.67E-04 |
| *VGF* | 1.95 | 1.08E-15 | *MPPED1* | -1.4 | 1.97E-07 |
| *PDE2A* | 1.94 | 8.76E-07 | *SGCE* | -1.37 | 7.10E-07 |
| *SLC14A1* | 1.90 | 1.26E-06 | *ZNF594* | -1.36 | 1.59E-06 |
| *GPR101* | 1.90 | 2.41E-15 | *PDE11A* | -1.36 | 1.16E-10 |
| *CTH* | 1.86 | 2.81E-09 | *MN1* | -1.35 | 1.92E-04 |
| *BDKRB2* | 1.86 | 1.26E-06 | *SLC26A6* | -1.34 | 1.32E-06 |
| *STC1* | 1.85 | 2.87E-09 | *IL11RA* | -1.34 | 1.44E-08 |
| *OR1I1* | 1.85 | 1.82E-18 | *SFTA3* | -1.33 | 2.23E-05 |
| *BEST1* | 1.84 | 7.87E-10 | *NRBP2* | -1.32 | 1.08E-03 |
| *NR1D1* | 1.83 | 1.16E-07 | *ASB16* | -1.3 | 6.16E-05 |
| *SEZ6L* | 1.82 | 1.68E-02 | *RASSF9* | -1.3 | 2.46E-03 |
| *TNFRSF10B* | 1.80 | 2.22E-05 | *MPP4* | -1.3 | 1.50E-02 |
| *DUSP4* | 1.80 | 1.33E-07 | *GRP* | -1.29 | 5.11E-07 |
| *H19* | 1.80 | 7.02E-14 | *FZD4* | -1.29 | 1.51E-07 |
| *SLFN11* | 1.78 | 6.02E-07 | *TSPAN2* | -1.29 | 1.21E-02 |
| *FAM129A* | 1.77 | 4.69E-07 | *SPTSSB* | -1.29 | 1.85E-05 |
| *RPL23AP82* | 1.76 | 1.67E-06 | *NRTN* | -1.29 | 4.21E-05 |
| *KLF6* | 1.75 | 1.90E-11 | *FAM172A* | -1.29 | 2.79E-07 |
| *FHL2* | 1.75 | 1.60E-08 | *LSS* | -1.29 | 7.98E-06 |
| *S100A14* | 1.74 | 2.51E-05 | *CBFB* | -1.28 | 1.61E-07 |
| *TMEM74* | 1.74 | 7.16E-03 | *MTSS1* | -1.27 | 8.59E-06 |
| *PDLIM3* | 1.73 | 6.73E-14 | *MYRFL* | -1.26 | 2.03E-04 |
| *ABCC6\|ABCC6P2* | 1.72 | 9.78E-03 | *PLXND1* | -1.26 | 5.99E-09 |
| *IL6R* | 1.71 | 4.10E-09 | *MUM1L1* | -1.26 | 2.08E-04 |
| *CEACAM6* | 1.71 | 5.99E-13 | *CD302\|LY75-CD302* | -1.26 | 1.53E-07 |
| *CRABP2* | 1.69 | 7.39E-04 | *ZNF521* | -1.25 | 1.40E-04 |
| *CHODL-AS1* | 1.69 | 1.49E-09 | *GABRG1* | -1.25 | 1.28E-02 |
| *RGS4* | 1.65 | 3.89E-14 | *PNRC1* | -1.24 | 1.06E-03 |
| *GJB3* | 1.65 | 7.22E-03 | *LINC00667* | -1.24 | 3.01E-03 |
| *BZW1* | 1.65 | 2.20E-08 | *IL1RAP* | -1.23 | 4.59E-02 |
| *ABCA12* | 1.64 | 1.09E-06 | *MEOX2* | -1.23 | 6.65E-04 |
| *KRT86* | 1.64 | 1.69E-03 | *PLA2G10* | -1.23 | 2.38E-07 |
| *LHX3* | 1.63 | 2.21E-10 | *IMMP1L* | -1.22 | 6.93E-07 |
| *MALL* | 1.63 | 3.46E-04 | *SIRT5* | -1.22 | 1.95E-02 |
| *DDIT3* | 1.62 | 4.93E-07 | *MST1\|MST1P2* | -1.21 | 4.55E-06 |
| *ANKRD22* | 1.62 | 5.01E-05 | *GSAP* | -1.21 | 1.86E-02 |
| *BCAT1* | 1.62 | 2.59E-05 | *PARP6* | -1.2 | 1.44E-04 |
| *PHGDH* | 1.60 | 8.40E-11 | *MTURN* | -1.2 | 1.17E-05 |
| *GOLGA7B* | 1.60 | 7.22E-06 | *TRPC4* | -1.19 | 1.28E-04 |
| *YOD1* | 1.59 | 7.80E-03 | *COL4A4* | -1.19 | 9.35E-03 |
| *ID3* | 1.59 | 3.51E-17 | *MAN2A2* | -1.18 | 2.35E-04 |
| *PHLDA1* | 1.59 | 3.33E-08 | *C6orf120* | -1.18 | 2.89E-03 |
| *BCAN* | 1.59 | 3.06E-05 | *CTGF* | -1.18 | 4.63E-05 |
| *KCNE4* | 1.58 | 3.32E-08 | *SEC62* | -1.18 | 5.44E-05 |
| *TNFRSF21* | 1.58 | 1.69E-10 | *NMU* | -1.17 | 2.13E-05 |
| *KRT19* | 1.57 | 1.05E-06 | *ST18* | -1.17 | 3.92E-06 |
| *CTSV* | 1.57 | 4.08E-08 | *SAMD9* | -1.17 | 3.49E-03 |
| *SPX* | 1.57 | 9.88E-07 | *SLC38A11* | -1.17 | 1.11E-05 |
| *MTRNR2L3* | 1.56 | 1.49E-06 | *ACADSB* | -1.16 | 2.69E-02 |
| *S100A14* | 1.56 | 4.55E-03 | *ANOS1* | -1.15 | 1.22E-02 |
| *PLA2G4A* | 1.55 | 5.14E-04 | *LYNX1* | -1.14 | 5.37E-08 |
| *RND3* | 1.55 | 6.33E-05 | *GALK2* | -1.13 | 3.44E-05 |
| *ANXA2* | 1.55 | 2.01E-02 | *VANGL2* | -1.13 | 3.37E-03 |
| *BACH1* | 1.54 | 6.63E-08 | *JAM3* | -1.13 | 1.27E-04 |
| *LOC101929959* | 1.53 | 2.41E-08 | *LINC00951* | -1.13 | 1.22E-06 |
| *JUN* | 1.51 | 6.83E-10 | *LIPA* | -1.12 | 2.32E-06 |
| *PKIB* | 1.51 | 3.99E-07 | KRBA2 | -1.12 | 3.20E-06 |
| *FGF21* | 1.50 | 4.82E-03 | VAT1L | -1.11 | 4.31E-05 |
| *CALCB* | 1.50 | 2.10E-09 | ZNF83 | -1.11 | 9.87E-05 |
| *AGR2* | 1.50 | 1.01E-05 | SMARCA1 | -1.11 | 5.80E-05 |
| *CSRNP1* | 1.50 | 7.39E-03 | CENPJ | -1.11 | 8.42E-05 |
| *MLPH* | 1.50 | 4.31E-09 | CYP4F2\|CYP4F3 | -1.1 | 8.04E-04 |
| *DNAJC22* | 1.48 | 3.46E-03 | GLRA3 | -1.1 | 1.43E-05 |
| *ATP6V0A4* | 1.48 | 2.05E-03 | MCF2L2 | -1.1 | 5.68E-03 |
| *LGALS1* | 1.48 | 3.02E-06 | C6orf163 | -1.1 | 3.50E-02 |
| *POTEF\|POTEJ\|POTEI\|POTEM\|POTEE* | 1.48 | 3.32E-05 | GPC4 | -1.1 | 6.94E-05 |
| *KRTAP5-9\|KRTAP5-8\|KRTAP5* | 1.46 | 5.15E-03 | FCHO2 | -1.1 | 1.11E-04 |
| *SMOC1* | 1.46 | 2.15E-05 | LOC155060 | -1.09 | 2.26E-03 |

**Table S4 (related to Fig. 4A).** Genes upregulated or downregulated in liver metastatic PNETs listed in datasets GSE73338 (comparing gene expression profiles between metastatic and non-metastatic PNETs) and GSE73339 (comparing gene expression profiles between primary and liver metastatic PNETs) were designated as PNET liver metastasis-related genes.

| **Up-regulated genes** | | | **Down-regulated genes** | | |
| --- | --- | --- | --- | --- | --- |
| **Gene name** | **GSE73338, fold change** | **GSE73339, fold change** | **Gene name** | **GSE73338,**  **fold change** | **GSE73339,**  **fold change** |
| *AGR2* | 1.491698 | 0.9303072 | *PCSK2* | -0.4880582 | -1.41739 |
| *CTH* | 0.336863 | 0.9970219 | *ZNF521* | -0.6963935 | -1.25196 |
| *GMDS* | 0.9026913 | 0.8899114 | *MAFB* | -0.8055404 | -0.50606 |
| *TACC2* | 0.642137 | 0.7868974 | *GUCY1A3* | -0.4953512 | -0.85299 |
| *KRT19* | 0.2332717 | 0.9839671 | *ANTXR1* | -0.2003208 | -0.81411 |
| *UBE2G1* | 0.0680698 | 0.37844 | *CTSO* | -0.1647367 | -1.03234 |
| *PHGDH* | 0.1530852 | 0.9999474 | *ZNF521* | -0.0632268 | -1.25196 |
| *SMOX* | 0.2183602 | 1.2077341 | *VAT1L* | -0.7669858 | -0.49683 |
| *SQSTM1* | 0.3571771 | 0.3905082 | *C1orf116* | -0.727641 | -0.40333 |
| *ATF5* | 0.3554002 | 0.8902845 | *FST* | -0.3257159 | -0.15006 |
| *DIAPH3* | 0.188204 | 1.2214642 | *FST* | -0.3257159 | -0.15006 |
| *TFF1* | 0.2336928 | 0.7947169 | *CTGF* | -0.339 | -0.76854 |
| *DDIT4* | 0.0833229 | 0.4624775 | *SGCE* | -0.289919 | -0.61257 |
| *DDIT3* | 0.0871793 | 0.6111815 | *FAM13C* | -0.4530656 | -0.46699 |
| *TNFRSF10B* | 0.2818599 | 0.3436757 | *RGS5* | -0.2749658 | -0.85749 |
| *MORF4L2* | 0.2536199 | 0.6297176 | *RGS5* | -0.2731794 | -0.85749 |
| *ADAMTS9* | 0.0642576 | 0.7160391 | *ETV1* | -0.1912649 | -0.62333 |
| *SERPINB5* | 0.2666517 | 0.6307697 | *GLRA3* | -0.2063701 | -0.83345 |
| *BDKRB2* | 0.2327451 | 0.2809825 | *ZNF83* | -0.4196427 | -0.59421 |
| *HN1* | 0.2148698 | 0.7099579 | *TMTC1* | -0.4500847 | -0.59481 |
| *ECE2* | 0.1958575 | 0.2428213 | *ZNF83* | -0.3967485 | -0.59421 |
| *ILF3* | 0.4027546 | 0.3899341 | *MN1* | -0.6152003 | -0.17545 |
| *STC2* | 0.2047847 | 0.5299268 | *FST* | -0.3257159 | -0.15006 |
| *FAM46A* | 0.4529588 | 0.2171354 | *FST* | -0.3257159 | -0.15006 |
| *FAM129A* | 0.3688353 | 0.1115557 | *TRERF1* | -0.0959734 | -0.75451 |
| *ANKS1B* | 0.1123023 | 0.6823734 | *TRERF1* | -0.084563 | -0.75451 |
| *RND3* | 0.2265444 | 0.2946618 | *TRPC1* | -0.3871666 | -0.50425 |
| *IGFBP2* | 0.0433711 | 0.6369344 | *IL11RA* | -0.1933275 | -0.45918 |
| *MLPH* | 0.3867637 | 0.1301568 | *LRRN1* | -0.4167375 | -0.36479 |
| *PKIB* | 0.0823099 | 0.4164619 | *OR51E1* | -0.3516477 | -0.22199 |
| *SNX5* | 0.0435265 | 0.5543594 | *COL4A4* | -0.4737107 | -0.1611 |
| *LMNB1* | 0.1765797 | 0.4872051 | *ZMAT4* | -0.2941379 | -0.41381 |
| *KSR1* | 0.3779269 | 0.2265104 | *SAMD9* | -0.1338396 | -0.49349 |
| *CBX2* | 0.4648881 | 0.0046084 | *CLNK* | -0.1469022 | -0.49319 |
| *ABLIM3* | 0.2811743 | 0.1956609 | *MTSS1* | -0.2070846 | -0.31652 |
| *BACE2* | 0.2512707 | 0.2321067 | *SYNPO* | -0.1597028 | -0.30898 |
| *BZW1* | 0.0727114 | 0.2917439 | *FGF11* | -0.0788997 | -0.51256 |
| *PINX1* | 0.2470201 | 0.3272542 | *MEOX2* | -0.0365682 | -0.466 |
| *CEBPG* | 0.1422845 | 0.3662371 | *TRERF1* | -0.0959734 | -0.44985 |
| *RRP7A* | 0.4128985 | 0.1642197 | *TRERF1* | -0.084563 | -0.44985 |
| *AREG* | 0.054271 | 0.1368642 | *SUSD4* | -0.0759278 | -0.3036 |
| *UGP2* | 0.3921699 | 0.0316533 | *FAM172A* | -0.2531295 | -0.18001 |
| *KRT6A* | 0.1639365 | 0.1105555 | *MTSS1* | -0.1147875 | -0.31652 |
| *LONP1* | 0.1269742 | 0.2526263 | *MBNL3* | -0.159682 | -0.24968 |
| *DCK* | 0.1153801 | 0.2022896 | *IFT74* | -0.238055 | -0.28282 |
| *MRPL44* | 0.0755486 | 0.3550908 | *TRPC4* | -0.0485771 | -0.37223 |
| *AXIN1* | 0.3828301 | 0.0345811 | *GPR39* | -0.3011654 | -0.1114 |
| *SLC35A2* | 0.0491456 | 0.2778887 | *SLC40A1* | -0.3875843 | -0.07951 |
| *SVIL* | 0.1351536 | 0.2275912 | *DDX60L* | -0.1446294 | -0.23654 |
| *WIPI1* | 0.1482562 | 0.1964673 | *ZNF346* | -0.2600658 | -0.12143 |
| *HEY1* | 0.2797044 | 0.0513364 | *ATP7A* | -0.033334 | -0.27852 |
|  |  |  | *YTHDC1* | -0.0609426 | -0.34711 |
|  |  |  | *FZD4* | -0.017702 | -0.29471 |
|  |  |  | *YTHDC1* | -0.0381343 | -0.34711 |
|  |  |  | *SLC40A1* | -0.2722892 | -0.07951 |
|  |  |  | *SLC16A10* | -0.2309174 | -0.10154 |
|  |  |  | *CASD1* | -0.0774584 | -0.24198 |

**Table S5 (related to Fig. 4A).** Genes listed in both the CAF signature and the PNET liver metastasis signature were designated as CAF-induced PNET liver metastasis-related genes.

| **Names of CAF-induced-PNET-liver-metastasis-related genes** | |
| --- | --- |
| *AGR2* | *Homo sapiens* anterior gradient 2 (AGR2) |
| *KRT19* | "*Homo sapiens* keratin 19, type I (KRT19)" |
| *SFRP1* | *Homo sapiens* secreted frizzled-related protein 1 (SFRP1) |
| *CTH* | *Homo sapiens* cystathionine gamma-lyase (CTH) |
| *GMDS* | "*Homo sapiens* GDP-mannose 4,6-dehydratase (GMDS)" |
| *TACC2* | "*Homo sapiens* transforming, acidic coiled-coil containing protein 2 (TACC2)" |
| *UBE2G1* | *Homo sapiens* ubiquitin-conjugating enzyme E2G 1 (UBE2G1) |

**Table S6.** PCR primer sequences.

| **Primer** | **Sequence (5’-3’)** |
| --- | --- |
| *Human ACTA2* | Forward: GCGTGGCTATTCCTTCGTTACTA |
|  | Reverse: GCATAGAGGTCCTTCCTGATGTC |
| *Human CTGF* | Forward: GCACCAGCATGAAGACATACCG |
|  | Reverse: GTTGTAATGGCAGGCACAGGTC |
| *Human IL1a* | Forward: GCATGGATCAATCTGTGTCTCTG |
|  | Reverse: CCTCTGAGTCATTGGCGATGG |
| *Human LIF* | Forward: AGTGCCAATGCCCTCTTTATTCT |
|  | Reverse: GGTGCCAAGGTACACGACTATG |
| *Human CCL2* | Forward: CTCATAGCAGCCACCTTCATTCC |
|  | Reverse: TCACAGCTTCTTTGGGACACTTG |
| *Human IL6* | Forward: TGCTTCCAATCTGGATTCAATG |
|  | Reverse: GGTTGGGTCAGGGGTGGTTATT |
| *Human CYCLOPHILIN* | Forward: ATACGGGTCCTGGCATCTTGTC |
|  | Reverse: GGTGATCTTCTTGCTGGTCTTG |
