# Supplementary figures and images for: Unraveling the impact of cancer-associated fibroblasts on hypovascular pancreatic neuroendocrine tumors
Source: Br J Cancer. 2024 Feb 10;130(7):1096–108. doi: 10.1038/s41416-023-02565-8 (PMC10991442; doi:10.1038/s41416-023-02565-8)

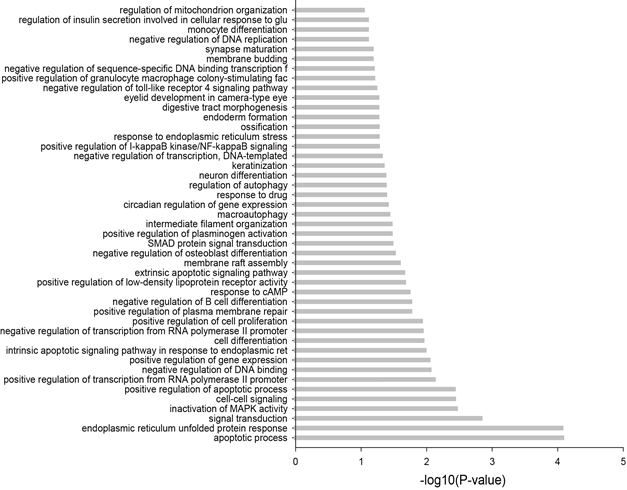

Supplement: Supplementary file 2 — Supplementary Figure 1 [file 41416_2023_2565_MOESM2_ESM.tif]
